# Supplementary material for: Emergency dispatchers as instructors of laypersons in unplanned out-of-hospital deliveries - Interdisciplinary qualitative study
Source: PLoS One. 2025 Jul 30;20(7):e0327808. doi: 10.1371/journal.pone.0327808 (PMC12310006; doi:10.1371/journal.pone.0327808)
Supplement: S1 Appendix — Emergency dispatchers’ (ED) frame stories. (DOCX) [file pone.0327808.s001.docx]

## S1 APPENDIX 1

Emergency dispatchers’ (ED) frame stories: 1. well script (WSED) and 2. unwell-script (UWSED)

1. *Imagine that it’s 2030 and there are ten maternity hospitals left in Finland. A layperson has performed an out-of-hospital delivery without the presence of emergency care or a midwife, and you participated in carrying out the delivery in the role of the emergency dispatcher. You feel that the situation went as well as it should: the mother and the newborn are well. Tell us a little story about how it went. What factors affected the situation?*
2. *Imagine that it’s 2030 and there are ten maternity hospitals left in Finland. A layperson has performed an out-of-hospital delivery without the presence of emergency care or a midwife, and you participated in carrying out the delivery in the role of the emergency dispatcher. You feel that the situation did not go as well as it should have: the mother and/or the newborn are not well. Tell us a little story about how it went. What factors affected the situation?*
