# Supplementary material for: Emergency dispatchers as instructors of laypersons in unplanned out-of-hospital deliveries - Interdisciplinary qualitative study
Source: PLoS One. 2025 Jul 30;20(7):e0327808. doi: 10.1371/journal.pone.0327808 (PMC12310006; doi:10.1371/journal.pone.0327808)
Supplement: S2 Appendix — Semi-structured interview framework (LP). (DOCX) [file pone.0327808.s002.docx]

## S2 APPENDIX 2

Semi-structured interview framework (LP)

First: orientation / ice breaker introduction, understanding the neutral language used by the interviewee

–strengthening trust

–shifting the power to the interviewee

Background information:

–the interviewee’s self-image

–relationship to the person giving birth

–previous experience of deliveries

–preparing for this delivery

Theme 1: unplanned out-of-hospital delivery

specifying the theme

–the day of the unplanned out-of-hospital delivery

–what happened, what happened then

–reasons, decisions made, participants

–positive or negative outcome

details

–resolution of the situation

–the wellbeing of the person giving birth and the newborn

–place of delivery

Theme 2: the emergency dispatcher

specifying the theme

–forming, maintaining and ending the connection (communication)

–the script of the emergency call (risk assessment): relevant instructions and disturbances

–expertise

–innovations (e.g. consulting a midwife)

–emotion work

–structure of the activity system

details

–positive and negative aspects

Theme 3: person giving birth

specifying the theme

–interaction

–object of activity

–emotion work

details

–means of communication

–interpersonal relationship

Theme 4: the layperson’s childbirth experience

specifying the theme

–feeling of safety and control

–positive/negative overall experience

details

–what kind of experience?

–ability to function

–calmness

–fear of complications

Theme 5: debriefing

specifying the theme

–emotion work (processing the experience)

–effect on close relationships

–innovations

details

–the most significant thing

–greetings to political decision-makers
